# Regulation of Intestinal Inflammation by Soybean and Soy-Derived Compounds

**DOI:** 10.3390/foods10040774

**Published:** 2021-04-04

**Authors:** Abigail Raffner Basson, Saleh Ahmed, Rawan Almutairi, Brian Seo, Fabio Cominelli

**Affiliations:** 1 Division of Gastroenterology & Liver Diseases, School of Medicine, Case Western Reserve University, Cleveland, OH 44106, USA; fabio.cominelli@uhhospitals.org; 2 Digestive Health Research Institute, University Hospitals Cleveland Medical Center, Cleveland, OH 44106, USA; sxa781@case.edu (S.A.); bys5@case.edu (B.S.); 3 Department of Pathology, School of Medicine, Case Western Reserve University, Cleveland, OH 44106, USA; raa140@case.edu

**Keywords:** inflammatory bowel disease, isoflavone, bioactive compound, isoflavones, inflammation, Crohn’s disease, western diet, plant-based

## Abstract

Environmental factors, particularly diet, are considered central to the pathogenesis of the inflammatory bowel diseases (IBD), Crohn’s disease and ulcerative colitis. In particular, the Westernization of diet, characterized by high intake of animal protein, saturated fat, and refined carbohydrates, has been shown to contribute to the development and progression of IBD. During the last decade, soybean, as well as soy-derived bioactive compounds (e.g., isoflavones, phytosterols, Bowman-Birk inhibitors) have been increasingly investigated because of their anti-inflammatory properties in animal models of IBD. Herein we provide a scoping review of the most studied disease mechanisms associated with disease induction and progression in IBD rodent models after feeding of either the whole food or a bioactive present in soybean.

## 1. Introduction

The burden of inflammatory bowel diseases (IBD), Crohn’s disease (CD) and ulcerative colitis (UC), are on the rise globally and represent one of the most prevalent chronic inflammatory conditions, particularly in the United States. Nearly half of Americans suffer from one or more chronic diseases, accounting for nearly 75% of aggregate healthcare spending [1].

Numerous lines of evidence indicate that alterations in the gut microbiota, shifting toward a pro-inflammatory state, plays a fundamental role in the development and progression of intestinal inflammation [2]. In this regard, the pathophysiology of IBD is attributed to a dysregulated T-helper cell immune response to gut microbiota catalyzed by the genetic susceptibility of an individual, which leads to a progressive and chronic loss of epithelial barrier integrity. As a result, intestinal microbiota and dietary antigens can easily translocate across the mucosal barrier and trigger mucosal immunity in the lamina propria, which serves to perpetuate the ongoing inflammatory response and chronic inflammatory state. Given the close relationship between inflammation and the generation of free radical species, oxidative stress has also been proposed as a potential underlying mechanism in IBD pathogenesis [3].

Although the causative factors of IBD remain unclear, disease incidence has been, in part, attributed to environmental factors, of which diet is considered the most important, associated with influencing the gut microbiota composition and, in turn, disease severity and progression. For instance, two major types of bacterial metabolites, short-chain fatty acids (SCFAs) and secondary bile acids, are known for their role in immune modulation, each causing opposing effects on intestinal inflammation at chronically high physiological levels [4]. Therefore, dietary management has been increasingly investigated for its therapeutic potential in IBD, focused on its ability to modulate the functional profile of gut microbiota and promote a balanced immunological response. Current approaches have focused primarily on chemically defined elemental diets (e.g., exclusive enteral/parenteral nutrition) or the restriction of specific food items (e.g., specific carbohydrate diet) [5,6]. However, the efficacy of such diets has been largely limited to pediatric populations [6]. More recently, plant-based diets, including the anti-oxidative agents contained therein, which interfere with cellular oxidative stress and cytokine production, have been among the dietary modalities investigated for their therapeutic potential in IBD [7,8]. With respect to individual food items, a growing body of preclinical evidence indicates that soybean, as well as soy-derived bioactives (e.g., isoflavones), have potent anti-inflammatory/anti-oxidant activity and can mitigate inflammatory changes in the gut induced either chemically or by diet (e.g., high-fat diet; HFD) [9,10,11]. In our own recent work, we demonstrated that soy, as a plant-based substitute for animal-based protein, in the context of an American diet (designed to mirror the NHANES survey), exerted a remarkable anti-inflammatory effect in the treatment and prevention of chronic CD-ileitis in mice genetically predisposed to CD [12].

Soybean or soya bean (*Glycine max*) is a species of legume native to East Asia, that today serves as an economically important crop in Western countries by providing a source of good-quality protein for both animals and humans. Soybeans are an exceptional source of essential nutrients, especially for protein and bioactive proteins (e.g., Bowman-Birk inhibitor; BBI), lipids such as monounsaturated fatty acids (oleic acid), and polyunsaturated fatty acids (PUFAs, n-3; α-linolenic acid, n-6; linoleic acid), as well as soluble and insoluble carbohydrates (e.g., raffinose, cellulose, pectin). Several lines of evidence from human and animal studies support the notion that high soybean intake provides significant health benefits, including the prevention of heart disease [13,14] and certain cancers [15,16,17]. However, soy also contains a unique mixture of ~139 phytochemicals (e.g., isoflavones, phytosterols, saponins) that are known to confer health benefits, many of which hold strong therapeutic potential in IBD [10,17].

Owing to the experimental advantages of animal models, particularly in the context of well-controlled dietary settings, we focus this review on the current understanding of how soybean can influence intestinal biology and inflammation, focusing on animal models, with validation from cell lines. Herein, we provide a scoping review of the major macronutrient, bioactive, and phytochemical components of soybean and their role in IBD, followed by the most studied disease mechanisms associated with disease induction and progression in IBD rodent models after feeding of either the whole food or a bioactive present in soybean.

## 2. The Bioactive Composition of Soy and Its Effect in Experimental IBD

Numerous studies have demonstrated the various health benefits of soy products in preventing heart disease, obesity, cancer, diabetes, osteoporosis, and regulating blood pressure and menopause symptoms [10,18]. Based on this evidence, in 1999, the Food and Drug Administration (FDA) authorized the “Soy Protein Health Claim” that 25 g of soy protein per day may reduce the risk of heart disease (Available at: https://www.fda.gov/media/108701/download, accessed on 5 March 2021). Today, the global soybean market is valued at around $148 billion as of 2018 and is projected to grow with a CAGR of 4% during the period of 2019–2025 [19]. During the last decade, soybean and soybean bioactives have been increasingly investigated because of their anti-inflammatory properties in animal models of IBD [10,20]. Below we provide an overview of the bioactive compounds relevant to the macronutrient (lipid, carbohydrate, protein) and bioactive composition of soybean in the context of their effects in IBD. We summarize the beneficial effects of soy and the bioactive compounds derived from soy in context to gut inflammation in Table 1.

### 2.1. Soy Lipid Fraction

Soy lipids comprise 20% of its total weight, of which 46–64% is constituted by PUFAs. Soybean is rich in fatty acids, primarily the omega-6 (n-6) PUFA linoleic acid (LA, C18:2) bound to different types of phospholipids and which constitutes ~55% of soybean oil. Other fatty acids present in soybean oil include palmitic (C16:0), stearic (C18:0), oleic (C18:1), and linolenic (C18:3) [21,22].

The effect of dietary fat on inflammation in IBD depends on the type, and amount of dietary fat consumed [23]. Current evidence suggests that the ratio between dietary omega-3 (n-3) and n-6 PUFA intake is directly linked to the pathology of inflammation-mediated human diseases such as IBD, obesity, cancer, atherosclerosis, rheumatoid arthritis [24]. For instance, diets high in saturated fat, particularly milk-fat, and/or excessive n-6 PUFAs (e.g., Western diet) exert a pro-inflammatory effect in IBD, the latter serving as a substrate for the production of pro-inflammatory prostaglandins, leukotrienes, and thromboxanes. By contrast, n-3 fatty acids, namely alpha-linoleic acid (ALA; C18:3, n-3; plant oils), eicosapentaenoic (EPA; C20:5, n-3), and docosahexaenoic acid (DHA; C22:6, n-3) are generally considered anti-inflammatory, serving to displace arachidonic acid and decrease inflammatory response severity [25]; albeit findings have varied [23].

In this context, raw soybean oil is not considered as an anti-inflammatory therapeutic dietary supplement because of its fairly high saturated fat content and high n-6 to low n-3 PUFA content (7:1 ratio) [26]. However, other bioactive compounds, including phospholipids and soyasaponins contained within the lipid fraction of soybean, have indeed been shown to exert anti-inflammatory effects [10].

#### 2.1.1. Phospholipids

Phospholipids in the diet are ingested in the form of glycerophospholipids, forming an important structural component and influencing the fatty acid composition and microstructure of cell membranes. Found in the yellow-brown fraction of the soybean, lecithin is considered a rich source of dietary glycerophospholipids, as well as phosphatidylcholine, which forms an important component of the intestinal mucus layer (along with protein mucins), that is essential to maintaining the barrier between gut microbiota and host intestinal mucosa [27]. Dietary phospholipids have been shown to reduce oxidative stress in the brain, reduce cardiovascular risks, and were shown to be effective in reducing inflammatory reactions in murine models [24,28,29]. However, it is the PUFA content of phospholipids, namely n-3 and n-6 PUFAs, which influence the nature of inflammatory responses primarily through the biosynthesis of phospholipid-derived lipid mediators that can have pro- or anti-inflammatory effects [24].

Phosphatidylcholine, a component in glycerophospholipids and mucus, is observed to have therapeutic applications in IBD [30]. This is spurred from the observation that patients with UC in remission have decreased levels of phosphatidylcholine in rectal mucus samples, which is consistent with the symptoms of weakened intestinal barrier typical in IBD patients [31]. Soybean phosphatidylcholine supplementation is reported to increase mucus secretion in the colon and improve mucus layer integrity [10]. As a vital component of mucus, phosphatidylcholine has a role in blocking hydrophobic bacteria and hydrophilic antigens from entering the intestine. These mucus-regenerating effects of phosphatidylcholine may additionally be effective for those who cannot accept traditional UC therapies, such as in the case of refractory UC [27]. Despite the potential therapeutic effect of phosphatidylcholine against UC, its pathophysiological mechanism for increased mucus secretion is unknown and requires further research.

#### 2.1.2. Soyasaponins

Also referred to as saponins, soybean soyasaponins are sterol or triterpene glycosides that have an oleanane-type aglycone with polysaccharide chains. To date, approximately 36 soyasaponins have been discovered and are classified into 4 groups based on their aglycones (soyasapogenols): Groups A, B, E, and DDMP

(2,3-dihydro-2,5-dihydroxy-6-methyl-4Hpyran-4-one) [32]. As aglycones of soyasaponins, soyasapogenols A, B, and E do not naturally occur in soybean but can be produced either via soyasaponin processing (and, therefore, may exist in soy products) or during endogenous digestion. Soyasaponins and soyasapogenols have been reported to have a protective effect against cancer and cardiovascular disease [32,33,34,35,36,37].

**Figure 1:**
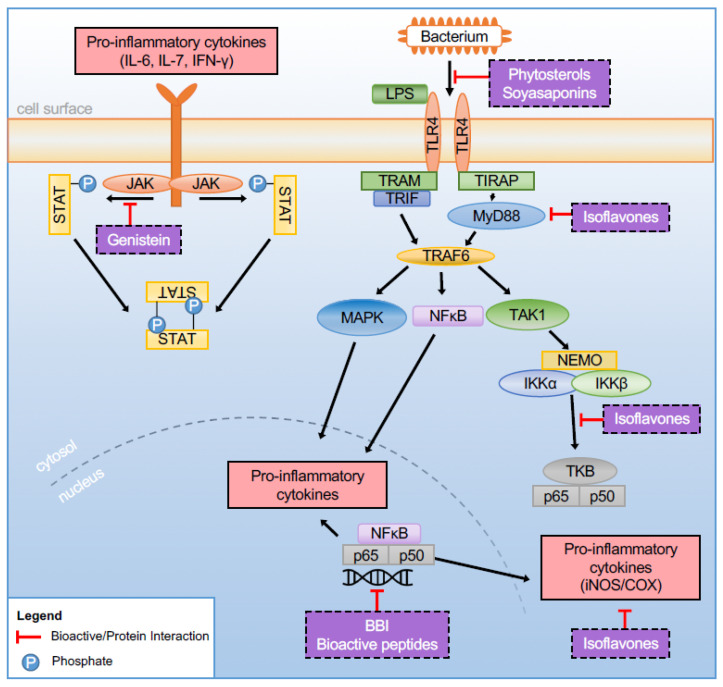
Soy-derived bioactive compounds mitigate pro-inflammatory signaling pathways JAK/STAT and TLR4/NF-κB. Soy-derived bioactive compounds such as phytosterols, soyasaponins, phytosterols, and Bowman–Birk protease inhibitors block key pro-inflammatory pathways JAK/STAT and TLR4/NF-κB, which are frequently upregulated in IBD.

While the physiological effects conferred by soyasaponins and soyasapogenols is dependent on their metabolism and absorption [32,38], both in vitro and animal studies have demonstrated their effect on gut health by exerting potent antioxidant, anti-inflammatory, and immunomodulatory activity (reviewed in [32]). Soyasaponin administration inhibited production of pro-inflammatory cytokine tumor necrosis factor-alpha (TNF-α), chemokine monocyte chemoattractant protein (MCP)-1, and the inflammatory mediators prostaglandin E2 (PGE2), nitric oxide (NO), cyclooxygenase (COX)-2, and inducible nitric oxide (iNOS) in lipopolysaccharide (LPS)-stimulated macrophages [39]. The anti-inflammatory activity of soyasaponin occurs through the inhibition of nuclear factor kappa B (NF-κB ) activation, a transcription factor widely associated with the onset and pathogenesis of inflammatory diseases [40,41] via the degradation of inhibitor of kappa B-alpha IκB-α [39,42,43]. Soyasaponins Ab, A1, A2, and I were found to contribute to these anti-inflammatory properties as well as inhibit iNOS and NF-κB activity in LPS-stimulated murine RAW 264.7 macrophages [43]. In peritoneal macrophages, soyasaponins have been shown to inhibit LPS binding to toll-like receptor-4 (TLR4) by regulating the TLR-4-mediated NF-κB activation [42] (Figure 1). In line with in vitro evidence, oral administration of soyasaponin was found to significantly decrease 2,4,6-Trinitrobenzenesulfonic acid (TNBS)-induced inflammatory markers, myeloperoxidase (MPO) activity, lipid peroxide, pro-inflammatory cytokines (interleukin-1-beta; IL-1β, interleukin-6; IL-6 and TNF-α expression) and NF-κB activation in the colon of mice, with concomitant increases in antioxidant enzymes [44].

Despite these promising findings for the therapeutic potential of soyasaponins in IBD, various factors require further investigation, namely, *(i)* the ability for intact soyasaponins to reach peritoneal macrophages in vivo, (*ii*) the bioactivity of soyasapogenols in enterocytes, and (*iii*) the safety of oral soyasaponin consumption in vivo [10].

#### 2.1.3. Phytosterols

Phytosterols are plant-derived sterols that are commonly found in the human diet. Although more than 100 types of phytosterols exist, the most common plant sterols, including those present in soybean (~300 mg/100 g of phytosterol), comprise of β-sitosterol, campesterol, stigmasterol, and ∆5-avenasterol [45,46]. Phytosterols have a similar structure and physiological function as that of cholesterol and are well-characterized for their ability to lower total cholesterol and low-density lipoprotein (LDL) levels, in which they affect bile acid homeostasis to reduce lipid absorption [47,48,49]. There is also evidence suggesting that colitis pathology can be mediated by phytosterol-induced T-cell changes, and that this effect may reflect the cholesterol-lowering properties of phytosterols due to the important role cholesterol metabolism plays in the activation of the adaptive immune response [50].

Besides the well-characterized effect on lipid profiles, in vitro [51,52] and in vivo [53,54], evidence is emerging that phytosterols also exert anti-inflammatory and anti-oxidative effects to reduce the inflammatory activity of immune cells [55,56]. In fact, both β-sitosterol and stigmasterol have been shown to elicit anti-colitic benefit in HFD- and chemically-induced colitis mouse models [54,57,58]. This occurs, in part, by suppression of NF-κB activation, with stigmasterol found to also downregulate COX-2 expression [58,59]. Stigmasterol also acts as an antagonist of farnesoid X receptor (FXR), a nuclear receptor responsible for maintaining intracellular bile acid homeostasis [60].

Other phytosterols, such as guggulsterone and phytosteryl ferulates (γ-oryzanol), have also been shown to attenuate acute colitis [49,61,62], although the exact anti-inflammatory mechanism of action remains unclear.

### 2.2. Soy Protein Fraction

Soybean comprised of ~35–40% protein based on the dry weight of a mature seed, is an easily digestible non-animal complete protein source (contains all nine essential amino acids, albeit low methionine content) that has a high Digestible Indispensable Amino Acid Score (DIAAS), on par with animal protein sources such as egg and dairy [21,63,64,65]. Because of this, soy has been used for decades in the food industry as an alternative protein and meat analogue and is one of the most used protein sources in many commercial laboratory rodent diets.

Beta-conglycinin and glycinin are the major source of soy proteins, accounting for ~65–80% of total proteins, and form the precursor of most peptides isolated from soybean. Of the various peptides, many have been shown to exert antioxidant, immunomodulatory, anticancer, antibacterial, angiotensin-converting enzyme (ACE)-inhibitory, and insulin-modulating activities (reviewed in [21]). Minor proteins in soy with bioactive properties in IBD include lunasin, lectin, and Bowman–Birk protease inhibitors [21].

#### 2.2.1. β–Sitosterol

β-sitosterol, the most prevalent phytosterol in soybeans, has been shown to have immunoregulatory properties. In peritoneal macrophages, β-sitosterol (10 and 20 µM) decreased phosphorylation of inhibitor of kappa B kinase-beta (IKK-β) and IκBα, two NF-κB intermediary proteins by directly inhibiting LPS binding to TLR4 [66] (Figure 1). Several animal models have also reported β-sitosterol to attenuate the severity of chemically induced colitis [49,57,67].

#### 2.2.2. β-Conglycinin and Glycin

Of the soy protein fraction, 90% is comprised of two storage proteins: β-conglycinin (7S globulin) and glycinin (11S globulin) [68]. These two storage proteins are significantly related to the allergenic effects of soy, as the two proteins remain stable when heated [69]. β-conglycinin is composed of α’, α, and β subunits, while glycinin is composed of five subunits that are a combination of acidic and basic parts [70].

Major storage proteins in soybean seeds, such as β-conglycinin, produce hydrolysates that have been shown to help maintain intestinal mucosa integrity [71,72], regulate intestinal flora balance, maintain intestinal health, and reduce the amount of enteric pathogen colonization [71,73]. In vitro studies also suggest that soybean glycopeptides (prepared from hydrolysates of β -conglycinin) inhibit enteropathogen adhesion, specifically *Escherichia coli*, *Salmonella typhimurium,* and *Salmonella enteritidis*, as well as prevent damage caused by bacterial infection to the plasma membrane of LoVo cells [74]. Further, high molecular weight β-conglycinin hydrolysates improved epithelial cell growth, and in Caco-2 cells, effectively increased transepithelial monolayer resistance (TER) and reduced the likelihood of *S. typhimurium* monolayer translocation [74]. In a dextran sodium sulfate (DSS)-induced intestinal mucosa injury model with female BALB/c mice, soybean β-conglycinin peptide treatment (50 or 500 mg/kg in 0.2 mL with 21.77% glutamic acid) for 28 days significantly reduced histological scores and MPO activity (indicative of neutrophil infiltration) in both protective and reparative settings compared to positive and negative controls [71]. Mechanistically, there was an inhibited expression of inflammatory factor NF-kB/p65.

Recent studies have correlated specific globulins with beneficial effects on metabolic disease. A β-conglycinin diet was shown to reduce atherosclerosis in mice, as well as reduce liver and plasma cholesterol in rats fed a high-fat diet (HFD) [75]. Dietary soy glycinin protein has also been shown to prevent muscle atrophy after denervation in mice [76]. A study on the anti-inflammatory activities of soy proteins on DSS-treated pigs showed that soy peptides exerted inhibitory effects on pro-inflammatory pathways mediated by T helper-1 (Th-1) type response, Th17, and upregulated FOXP3+ T-regulatory (T-reg) response in the colon and ileum [72]. There is also evidence that tripeptides derived from the enzymatic hydrolysis glycinin, specifically valine-proline-tyrosine (VPY), play a role in inhibiting the production of pro-inflammatory mediators and down-regulate the expression of pro-inflammatory cytokines. It is suggested that VPY may be a potential therapeutic agent for IBD by targeting the Human peptide transporter 1 (PEPT1), an uptake transporter in the small intestinal lumen [77,78,79], which is up-regulated during intestinal inflammation [77].

Soy-derived peptides have been shown in vivo to be effectively absorbed, suggesting their function as a transportable bioactive peptide [77,78,79,80]. In a DSS-colitis female BALB/C mice model, 2-week pretreatment of drinking water with transported tripeptide VPY (0.1 and 1 mg/mL) yielded anti-inflammatory effects by reducing colitis symptoms, weight loss, colon damage, MPO activity, and gene expression of colon pro-inflammatory cytokines (TNF-α, IL-6, IL-1β, interferon gamma; IFN-y, and IL-17) compared to positive and negative controls [77]. Additionally, in vitro, VPY (2 and 4 mM) showed anti-inflammatory effects by inhibiting IL-8 and TNF-α secretion from Caco-2 and THP-1 cells, respectively. In the presence of a competitive substrate Gly-Sar, VPY transport decreased, indicating that anti-inflammatory activity was mediated by PEPT1 [77].

#### 2.2.3. Lectin

First characterized by Peter Hermann Stillmark in 1888, lectins are a class of non-immune origin carbohydrate-binding proteins found in all eukaryotes and many bacterial species and viruses, and are widely distributed in grain legumes, including soybean. In plants, lectins have a defensive role against predators. Orally ingested lectin remains undigested in the gut and is able to bind to various cell membranes, including epithelial cells and glycoconjugates in intestinal and colonic mucosa. Lectin binding in the gut can negatively affect gut immune function, microbiota profiles in the gut, and damage mucosal cells [81].

Soybean agglutinins (SBA), also known as soybean lectins, are non-fiber carbohydrate-related proteins that represent 5–7% of the soybean and are considered the main anti-nutritional factors that affect the quality of soybean [82]. While various beneficial bioactive effects have been attributed to SBA (antitumor, antifungal, antiviral, and antibacterial activities) [83,84,85], SBA are also able to negatively affect gut health by disrupting gut barrier function [86,87], inducing local inflammatory responses [88,89], decreasing immunological responses [88], and interfering with the balance of the intestinal microbiota [82,90]. Modulation of gut microbiota by SBA occurs via three possible mechanisms, including; *(i)* SBA binds to small bowel epithelial cells resulting in alterations to the glycan structures of the intestinal mucosa and changes to bacterial binding sites, which in turn, selectively stimulates growth of some bacteria, *(ii)* SBA serves as a nutrient source for bacteria, and *(iii)* SBA induces alterations to the gut mucosal system resulting in reduced immunoglobulin A (IgA) secretion and inhibition of bacterial proliferation [82].

Intake of SBA by intestinal epithelial cells can also elicit a toxic effect in most animals [82]. In rats, intraperitoneal injection of SBA induced a dose-dependent inflammatory response, which was blocked by pretreatment with glucocorticoid or by co-injection of N-acetyl-galactosamine, but not other sugars [91]. Similarly, high-dose administration of SBA has been shown to increase intestinal permeability in pigs, whereas low-dose had no effect [92]. On the other hand, when present in circulating blood, soybean SBA has been shown to elicit an anti-inflammatory effect [91]. Despite the above deleterious effects of SBA, aqueous heat treatment, particularly pre-soaking soybean in water and cooking (212 °F, 100 °C for at least 10 min) [93], almost completely deactivates SBA, and thus the presence of SBA in human foodstuffs is relatively low [94,95].

#### 2.2.4. Lunasin

Lunasin is a naturally occurring 43-amino acid peptide with high concentrations of the soybean’s total aspartic acid content [96]. Beside soybean, lunasin is found in other beans, grains, and herbal plants, including wheat, barley, rye, sunberry, wonderberry, bladder-cherry, jimson weed, tofu, tempeh, whole wheat bread, at concentrations ranging from 0.013 to 70.5 mg protein lunasin/gm of protein [96,97]. Lunasin has been intensely investigated for almost two decades for its potential use as a dietary supplement given its rare composition and the unusual aspect of the polypeptides structure [96,98], which have various proposed health benefits, including anti-carcinogenic, anti-oxidant, and anti-inflammatory properties [99,100,101].

In vitro studies have demonstrated the ability of lunasin to suppress LPS-induced inflammatory reactions in macrophages via reduction of pro-inflammatory cytokines production (IL-6, TNF-α), as well as other pro-inflammatory mediators, such as PGE2, through modulation of COX-2, and iNOS/nitric oxide pathway by NF-κB pathway inhibition [101,102,103]. Lunasin has also been reported to inhibit the translocation of p50 and p65 subunits in NF-κB in the cell nucleus, thereby inhibiting gene transcription and production of pro-inflammatory molecules [102,103]. In a similar fashion, lunasin administration significantly attenuated the severity of DSS-induced inflammation, in part by decreasing colonic COX-2 expression [104], an important mediator of the inflammatory response that is known to increase in DSS-colitis [105,106].

While lunasin is heat stable and readily bioavailable [72,107], there is evidence to suggest that processing methods may affect lunasin efficacy. For example, commercial lunasin from soy was found more protective of DSS-induced inflammation in Swiss Webster mice compared to that of lunasin of soybean extract at higher doses. This suggests that lunasin purity and its anti-inflammatory properties are affected by method of lunasin extraction [104].

#### 2.2.5. Bowman–Birk Inhibitor (BBI)

The Bowman–Birk inhibitor is a serine protease inhibitor present as an anti-nutritional factor in soybean and various other types of legumes. BBIs resist digestion and have been shown to reach the small and large bowel intact [108,109], or be absorbed through the gut lumen and act systemically before being excreted in urine [110]. The trypsin-like and chymotrypsin-like proteases [111,112] of BBI are, however, thought to decrease protein digestibility [113] and possibly promote pancreatic disease. As such, BBI is inactivated in the processing of soy concentrate (e.g., soymilk) [10].

However, BBI is known to have anti-inflammatory activity in both in vitro and in vivo systems and has long been recognized as a potent inhibitor of malignant transformation across various cell lines [114,115]. BBI also exerts potent anti-inflammatory activity, particularly in the gut [116], acting to effectively inhibit both serine proteases released from inflammation-mediating cells [117], as well as suppress proteolytic and oxidative damage that occurs during inflammation [118]. Given that serine proteases are known for their active involvement in pro-inflammatory actions [117], and have been implicated in both the production of pro-inflammatory cytokines [119] and the aberrant inflammatory process in IBD [117], BBIs have been investigated for their therapeutic potential as a natural alternative to protease inhibitors.

In DSS-treated Swiss Webster mice, 0.5% of BBI concentrate supplementation before and after colitis induction resulted in a significant reduction in mortality (by 15%) and mortality scores (by 50%) compared to non-supplemented mice fed a standard diet [118]. The beneficial effects of BBI were, however, not seen when BBI was supplemented after DSS-colitis induction [118]. In line with these findings, feeding of a BBI-containing fermented soy germ extract was shown to significantly reduce TNBS-colitis in Wistar rats [120].

The role of BBI in regulating inflammation is in part via its ability to decrease LPS-induced pro-inflammatory cytokines (IL-1β, TNF-α, IL-6) and increase anti-inflammatory cytokine (IL-10) in macrophages [121,122], known mediators of inflammation and immune-activation. In addition, BBI has been shown to increase the proportion of CD4 + CD25+Foxp3+ Tregs, which exert immune-suppressive activities [123].

### 2.3. Soy Carbohydrate Fraction

The carbohydrate composition of soybean, comprising 9% dietary fiber from its total weight, consists mostly of oligosaccharides (‘soy oligosaccharides’), including stachyose, raffinose, sucrose, and common components formed by various linkages of mono- and oligosaccharides [124,125]. Raffinose and stachyose are non-digestible in the gut and thus remain intact until reaching the lower intestine, where they are metabolized by certain bacteria, which possess the alpha-galactosidase enzyme.

#### Soy Oligosaccharides

Several studies have investigated the prebiotic potential of soy oligosaccharides [126,127], particularly in terms of gut health. Overall, soy oligosaccharides have been shown to benefit immune function by promoting the abundance and metabolism of beneficial commensal gut bacteria [128], in part, via enhanced T-lymphocyte and lymphocyte proliferation [125]. Further, soybean meal oligosaccharides have been shown to promote competitive exclusion of pathogenic bacteria [129]. There is also evidence that soybean oligosaccharides influence hematological and immunological parameters, for instance, *(i)* by increasing levels of superoxide dismutase (SOD) and IgG, *(ii)* by promoting splenocyte proliferation, as well as enhancement of the number of antibody-forming cells in normal mice, and *(iii)* via attenuation of immune effects in SAM- and S180-treated mice [130].

In general, soybean oligosaccharides have been shown to increase microbial diversity in the gut, including the abundance in short chain fatty acid (SCFA)-producing bacterial taxa such as *Bifidobacterium* and *Lactobacillus* [124,125,131] (although soy protein intake has also been shown to exert similar effects in vivo) [132]. Unfortunately, bacterial metabolism of these oligosaccharides in the colon results in gas production, lowering acceptability of intake. Soy fermentation eliminates this problem as well as increases the bioavailability of soy isoflavones.

### 2.4. Soy-Derived Isoflavones

A class of phytochemicals, isoflavones, are plant-derived polyphenolic compounds found in a variety of legumes, including soy foods, with soybeans providing the richest source containing between 140–1530 mg/kg (vs. soy milk of 12–130 mg/kg) [133]. Of the 12 different of soybean-derived isoflavone isomers, the two major glycosidic forms associated with health benefits include: Daidzein (7-hydroxy-3-(4-hydroxyphenyl)-4H-chromen-4-one) and genistein (4′, 5, 7 Trihydroxyisoflavone), where they comprise 40% and 50% of total isoflavone composition, respectively [134].

The physiological effects of isoflavones depend on their bioavailability, with the bioavailability of genistein being greater than that of daidzein [135]. In the small intestine, *Bifidobacteria* and lactic acid bacteria that possess β-glucosidase activity are able to hydrolyze isoflavone glucosides into aglycones (reviewed in [136]). The derived metabolites therein are either absorbed by the host or metabolized further in the colon by colonic bacteria into metabolites of various estrogenic potential, such as equol, O-desmethylangolensin, and p-ethylphenol [137,138,139,140]. These metabolites are then absorbed via the portal vein and can persist in plasma for ~24 h [141]. Isoflavones have been reported for their beneficial effect in cardiovascular disease, osteoporosis, cancer, and alleviation of menopausal symptoms (reviewed in [142]).

Isoflavones are classified as phytoestrogens and exhibit both functional and structural similarities to the mammalian estradiol molecule, giving isoflavones their ‘estrogen-like’ activity via the estrogen receptor (ER) [140,143,144,145,146,147,148]. Although isoflavones bind to both α and β isoforms of ERs, there seems to be a preferential binding and activation of approximately 20 times towards ERβ than to ERα [143,144]. Notably, the predominant ER subtype expressed in colon tissues is ERβ, and it serves to maintain a normal epithelial architecture protecting against chronic colitis [149,150].

In recent decades, extensive epidemiological evidence, together with preclinical in vivo and in vitro studies, indicate that isoflavones also exert potent anti-inflammatory activity in a range of inflammatory diseases via increased antioxidative activities, NF-κB regulation, and reduced pro-inflammatory factors including enzymatic activity and cytokine levels (reviewed in [151,152]). The antioxidant activity of isoflavones is largely attributed to their inhibitory effect on the COX-2, an enzyme that mediates the conversion of arachidonic acid to pro-inflammatory prostaglandins. Prostaglandin is an important mediator in the inflammation process, and its synthesis is increased in inflamed tissues [153]. By comparison, raw soybean oil, which is void of isoflavones, has been shown to significantly raise the levels of arachidonic acid [154].

Numerous studies support the anti-oxidant and anti-inflammatory activity of soy isoflavones; however, their therapeutic role in human IBD remains largely unknown. Data from preclinical rodent studies suggest that the antioxidant activity of soy isoflavones occurs via the scavenging of free radicals, upregulation of antioxidant enzyme systems, and promotion of tight-junction protein expression and TLR4 signaling activity [149,150]. Diets containing high isoflavones contents showed consistent and significant elevation of antioxidant enzymes in various organs [155,156]. Fermented soy germ contains phytoestrogen that is similar to 17B-estradiol in women [150,157], which has been demonstrated to mitigate effects of IBD, such as decreased paracellular permeability and increased tight junction sealing [150,158]. In a partial restraint stress female Wistar rat model, the estrogenic and protease inhibitor properties of phytoestrogen-rich soy germ (34.7 µmol/gm of isoflavones vs. 17β-estradiol benzoate) were shown to prevent stress-induced intestinal hyperpermeability and hypersensitivity, although had no effect on plasma corticosterone [48].

#### 2.4.1. Genistein

Among the soybean isoflavones, genistein is considered the most predominant in the human diet [159]. Genistein has been shown to act as a potent agent in both the prevention and treatment of cancer and various chronic inflammatory diseases [160]. The anti-cancer activity of genistein is mainly attributed to its ability to mediate apoptosis, cell cycle, and angiogenesis, as well as inhibit metastasis [161,162]. Genistein has also been suggested to reduce obesity in adults due to its estrogenic activity on genes associated with regulation of lipolysis, lipogenesis, and adipocyte differentiation via ERβ, and 5′ adenosine monophosphate-activated protein kinase (AMPK) signaling within muscle and adipose tissue via ERɑ [162,163]. However, the effects in adipose tissue appear to be sex- and dose-dependent, and the effects of soy may vary based on time of administration (early development vs. adult), likely due to differences in ER expression and levels of endogenous estrogens [163].

In IBD, the anti-inflammatory properties of genistein have been reported both in vitro and in vivo. For example, at physiological concentrations (0.1 μM–5 μM), genistein was found to inhibit TNF-ɑ-induced endothelial and vascular inflammation in C57BL/6 mice via mediating protein kinase pathway A [164]. Other studies have also demonstrated genistein inactivation of NF-κB signaling [165,166]. Rodent models of chemically-induced colitis have also demonstrated marked attenuation of colitis severity and reduced pro-inflammatory cytokine profiles following genistein treatment [20,167,168]. In vitro administration of genistein in Caco-2 cells was further shown to improve both cell viability and cellular permeability and inhibited DSS-induced activation of TLR4/NF-κB signaling [167]. Further, genistein treatment was shown to skew M1 macrophages toward the M2 phenotype, marked specifically by increased expression of arginase-1 and reduced systemic cytokine profiles. Additionally, genistein increased the number of dendritic cells and IL-10 producing CD4+T cells, which in part attenuated colitis symptoms [168].

Another route in which isoflavones modulate intestinal inflammation in epithelial cells is via activation of the Janus kinase (JAK) signal transduction and activator of transcription (STAT) pathway via cytokine signaling. During inflammation, STAT activation is needed for CD4+ T-cell differentiation into the T-helper phenotypes (i.e., Th1, Th2, Th17) [169,170]. Dysregulated STAT phosphorylation is implicated in the development of chronic inflammation due to excessive T-helper cell response by inhibiting immune cell apoptosis [171]. In vitro, isoflavones have been shown to modulate JAK-STAT activity in intestinal epithelial cells. In murine macrophages, genistein was shown to inhibit STAT1 translocation induced by LPS [172]. In Caco-2 cells, low-dose genistein treatment (3 µM) decreased STAT3 translocation by 56%, whereas higher doses (30 µm) decreased STAT 1 nuclear translocation by 23% [173]. STAT inhibition by genistein and possibly other soy isoflavones is viewed as one of the main mechanisms of action of genistein in inflammatory diseases [173,174,175].

It is important to note that genistein (and flavonoids) is susceptible to oxidative degradation, particularly when exposed to oxygen, light, moisture, heat, and food processing conditions that might affect their nutraceutical value, and make them less active, and reduce their absorption efficiency [176,177,178]. In this regard, encapsulation systems, such as microencapsulation (e.g., obtained by water-soluble Chitosan obtained by Maillard reaction) and nanoencapsulation, could serve as useful tools for oral administration of genistein to preserve biological, antioxidant, and anti-inflammatory properties, as well as the functionality of genistein in vitro and in vivo [179,180,181,182].

#### 2.4.2. Equol

The soy isoflavone, equol, is a daidzein-derived metabolite that exists in two enantiomeric forms, R- equol and S-equol, the latter naturally metabolized by microbiota in the intestines of humans and rodents [183]. In the gut, S-equol is produced from daidzein via 21 different intestinal bacterial strains, which have been identified in 30–50% of the population. However, prevalence rates are lower (20–30%) in Westerners compared to that of Asians (50–80%) [184,185]. While studies from northeast Asia have suggested that S-equol, and not dietary soy, is inversely associated with incident coronary heart disease, various short-term RCTs have found that both soy isoflavones and S-equol improve arterial stiffness [186,187,188,189], an independent and important predictor of coronary heart disease [190].

Both clinical and experimental evidence shows that equol holds unique immune properties, having greater estrogenic and antioxidant activity compared to most other isoflavones [157,183,191,192]. In vitro, genistein, daidzein, and equol were shown to significantly decrease nitric oxide production via inhibition of iNOS mRNA expression and protein in a dose-dependent manner. In addition, pre-treatment of human intestinal cells (Caco-2 cell line) with the isoflavones reduced LPS-induced inflammatory responses via decreased NF-κB activation [193]. However, in a DSS-colitis female BALB/C mouse model comparing the effect of genistein, daidzein and equol, daidzein, and particularly equol were found to severely perpetuate the DSS-induced effects on body weight, resulting in 14% survival in equol-treated mice. It is important to note that effects were dose-dependent, with significant reductions in body weight seen at equol dosages of 20 mg/kg body weight and not at 2 or 10 mg [194]. Equol administration also resulted in decreased production of anti-inflammatory cytokine IL-10 by mesenteric lymph node T-cells. The worsening of colitis by equol was proposed to reflect T cell-dependent and -independent mechanisms, given the significantly lower survival rate seen in equol-treated severe compound immunodeficiency (SCID) mice compared to controls [194]. Notably, the consumption of purified isoflavones by equol producers results in significantly higher levels of equol in both urine and blood plasma, about 10 to 1000 times higher than that of non-producers fed the same supplement [195].

## 3. Mechanisms of Action

### 3.1. Intestinal Mucosa Permeability

The epithelial mucous layer consists of mucus glycoproteins (mucins or MUC;) and trefoil factors (TFF) secreted by goblet cells that collectively provide the first line of defense against pathogens, both of which are critical to protecting the intestine from inflammation [196,197]. Impairments in the gut epithelial barrier are characteristic of IBD and results in increased gut permeability to bacterial pathogens and other antigens, which in turn, triggers and perpetuates ongoing immune responses and chronic inflammation [198]. There are more than 40 different tight junction associated-proteins involved, with occludin, claudin, zonula-occludens-1 (ZO-1), and junction adhesion molecules (JAM) proteins considered the most important in maintaining epithelial integrity. These proteins are integral proteins associated with peripheral membrane proteins, such as ZO-1, which play a role in scaffolding and anchoring integral proteins [199,200].

Chemically-induced colitis studies have demonstrated the protective effects of soy isoflavones on colonic inflammation and specifically, intestinal barrier integrity, albeit alterations in the expression of tight junction protein appears to vary not only between the different isoflavones but also between the various fractions (isoflavones, proteins) found within soy. For instance, in DSS-treated female institute of cancer research (ICR) mice, supplementation of a standard rodent diet with 0.5% soy isoflavones significantly enhanced occludin colonic mRNA [149], whereas in DSS-treated female BALB/C mice, genistein supplementation (600 mg/kg diet) enhanced both ZO-1 and occludin colonic mRNA, as well as lowered serum LPS (as a marker of colonic permeability) following colitis induction [167]. The combination of a barley and soybean mixture enriched in β-glucans, genistein, and daidzein was shown to significantly prevent loss of tight junction proteins ZO-1, occluding, and claudin-1 in colonic epithelial cells of female C57BL/6 mice following DSS treatment. In vitro, the barley-soybean mixture dose-dependently recovered the DSS-induced loss of tight junction proteins within the monolayer of Caco-2 cells, an epithelial cell line broadly used as a model of intestinal barrier [201].

Protease-activated receptors (PAR) are a subfamily of the G-protein-coupled receptor family that are activated through the cleavage of a part of their extracellular domain. Among the PARs, PAR-2 is highly expressed on endothelial cells and has been widely studied for its ability to modulate inflammatory responses, with various proteases able to act as inflammatory mediators and disrupt barrier function, due to their ability to cleave and activate PAR [202,203,204,205,206,207]. Indeed, several studies have shown that patients with IBD have increased levels of protease activity in feces [204,206,207,208,209,210,211]. While the exact mechanism behind the regulation of fecal proteolytic activity is not fully understood in the context of IBD, soy protein, soy isoflavones, and soy-derived serine protease inhibitors (soy BBI) are promising candidates for its regulation. For example, the protease inhibitor activity of fermented soy germ extract containing both BBI and isoflavones (55% daidzein, 30% glycitein, and 15% genistein in aglycone forms) was shown to significantly reduce the severity of TNBS-induced colitis and was associated with increased intestinal permeability at 24 h and 3-days post colitis in Wistar rats [120]. Additionally, fermented soy germ prevented luminal increases in protease activity and decreased epithelial Protease-activated receptor-2 (PAR-2), independently of ER-ligand activity [120]. In a model of stress-induced irritable bowel syndrome (IBS), the same group found that fermented soy germ extract also prevented the decreases in occludin expression resulting from prolonged stress when compared to non-supplemented controls [150]. In both studies, however, the protective effects of soy germ extract were reversed via administration of the estrogen receptor antagonist ICI 182.780, suggesting that the protective properties are linked to ER-ligand activity [120,150].

The protective effects of soy on intestinal permeability are, however, not entirely dependent on ER-ligand binding activity of isoflavones. For example, supplementation with isoflavone-free soy protein concentrate was shown to attenuate the effects of DSS-colitis and prevent loss of gut barrier function in male CF-1 mice, suggesting that different components within soybean (other than isoflavones) exert protective effects on intestinal permeability [11]. Isoflavone-free soy protein supplementation also reduced colonic glucagon-like peptide-2 (GLP-2) protein levels (a key regulator of intestinal mucosa via regulation of epithelial cell growth) but had no effect on mRNA expression of claudin-1, occludin, or the ratio between the two [11]. In vitro, co-treatment with soy protein concentrate in Caco-2 cells mitigated both intracellular oxidative stress as well as DSS-induced increases in monolayer permeability. Intriguingly, hydrolysis of soy protein concentrate with pepsin and pancreatin (reducing thiol content) reduced the radical scavenging activity but not the effect on monolayer permeability, suggesting that the beneficial antioxidant effect and effect on permeability are a result of different underlying mechanisms of action or different components within soy protein [11]. By comparison, administration of soy protein BBI to DSS-treated male C57BL/6 mice was found to only increase colonic mRNA expression of occludin, whereas colitic mice treated with pea seed albumin extract (known to contain BBI) exhibited increased mRNA expression of MUC-3, occludin, and ZO-1 [212]. Treatment with the albumin fraction of pea seed extract, however, had no significant effect on tight junction protein expression [212].

The effect of soy protein on mucosal integrity may also vary based on the time of administration. For example, in DSS-colitis C57BL/6 mice fed from 7 weeks-of-age, either a soy-, casein-, or whey-based diet with or without the addition of probiotic *Lactobacilus rhamnosus GG*, colonic Muc1 but not Muc2 expression was significantly lower in soy-fed DSS mice. The addition of *L. rhamnosus GG* to the diet had no effect on colonic MUC expression [213]. By comparison, feeding a soy protein isolate-based AIN-93G diet for 21 days to 3-week-old C57BL/6 weanling mice resulted in a suppression of secretory IgA and mucin (decreased expression of Muc2, Tff3, GRp94 and Agr2) in the ileum compared to mice fed the referent casein-based diet [214].

Current data indicate that excess dietary fats (i.e., HFD) can promote LPS-induced gut barrier dysfunction, resulting in enhanced local/serum LPS levels, which, in turn, promotes intestinal inflammation, alterations in tight junction signaling, intestinal epithelial cell dysfunction, and hyperpermeability [215].

Emerging evidence indicates that various soy bioactives can attenuate the inflammatory activity induced by an HFD. For instance, in an HFD-induced obese Sprague Dawley rat model, soy isoflavone supplementation increased ZO-1 expression and reduced LPS concentrations when compared to that of non-supplemented HFD-fed mice, with increases in both occludin and Muc-2, found in mice supplemented with high doses of isoflavone [216]. In another study, dietary supplementation (21d) of either a high- or low-fat diet (30% vs. 6% fat) with tempe, a fermented soy product, resulted in markedly elevated fecal mucins (indices of intestinal barrier function) and IgA in male Sprague Dawley rats compared to non-supplemented rats [217]. In HFD-fed C57/BL6 mice supplemented with genistein for 6 months, mice exhibited lower circulating levels of LPS (vs. non-supplemented mice), suggesting a protective effect on mucosal gut barrier health (preventing LPS influx) [218]. Similar protective effects on intestinal permeability were reported in SAMP1/YitFC mice challenged with and without DSS-colitis and fed a Western-style diet [12]. In a recent RCT, combined supplementation of soy and vitamin D significantly reduced plasma inflammatory markers and fecal protease activity, as well as improved gut permeability in women with irritable bowel syndrome (IBS) [219].

Despite the protective effects on intestinal permeability observed from both the isoflavone and protein fraction of soy, such effects are not seen with soybean oil. In fact, a soybean oil-supplemented elemental diet increased intestinal permeability (measured by urinary excretion of phenolsulfonphthalien; PSP) and intestinal damage to a level comparable to that of standard chow-fed male Sprague Dawley rats following indomethacin-induced small bowel inflammation [220]. The pro-inflammatory effect of soybean oil compared to that of soybean may be attributed to the high n-6 to n-3 ratio, as well as the absence of isoflavones or other soy-derived peptides.

### 3.2. Oxidative Stress

Various lines of evidence suggest that the modification of macromolecules (e.g., DNA, lipid, protein) induced by reactive oxygen species (ROS) plays an important role in DNA damage, genotoxicity and carcinogenesis [221,222]. Overproduction of ROS and reactive nitrogen species are known to exacerbate symptoms in IBD, affecting the redox equilibrium within the gut mucosa. Antioxidant enzymes, for example, SOD, are capable of eliminating ROS and byproducts of lipid peroxidation, which, in turn, protects tissues and cells from oxidative damage. Recent studies show that soy isoflavones and protein concentrate are able to reduce radical scavenging activity [149,216].

High-fat diets are known to induce oxidative stress and lipid peroxidation, and reduce antioxidant enzyme activities in various organs of obese mice, particularly the intestinal mucosa [44]. Soy isoflavones, particularly at high dosages (450 mg/kg and 150 mg/kg), were shown to significantly attenuate HFD-induced intestinal oxidative stress in the colon via upregulation of important ROS scavengers, including SOD, total antioxidant capacity, glutathione peroxidase (GSH-Px), and catalase, with a concomitant reduction in malondialdehyde, a product of lipid peroxidation and an indicator of protein oxidative damage [216]. Significant reductions in free radical activity has also been reported from soy protein concentrate supplementation in a standard rodent diet in vivo, as well as in vitro, although pre-oxidation of soy protein concentrate or the blocking of free thiols abolished the latter effects [11]. In another study, soy isoflavone extract exhibited strong antioxidant activity in vitro. However, a dose-dependent response was observed, with antioxidant activity found to plateau at higher concentrations [155]. When administered to rats, soybean isoflavone extract (250 ppm) enhanced SOD activity in various organs (lungs, small intestine, kidney) when compared to vitamin E, with SOD activities found to markedly increase from the 8th to 16th week of feeding, and the most notable effects observed after 24 weeks [155]. Of interest, however, laboratory-prepared tofu (containing ~50 ppm isoflavones) had better effects than the soy extract (containing ~250 ppm), suggesting that that molecules other than isoflavones present in tofu (soybean-based product) may have a synergistic effect on in vivo induction of antioxidant enzyme activity [155].

Laboratory studies with genistein have yielded mixed results regarding oxidative stress. While some have shown that dietary supplementation significantly decreases expression of molecular and biochemical markers of inflammation [20,120] and increases antioxidant enzyme activity in various mouse organs [147], others have reported genistein to enhance colonic oxidative stress and colon carcinogenesis [223], with the combination of genistein and epigallocatechin-3-gallate, a green tea polyphenol, shown to enhance tumorigenesis in Apcmin/+ mice [221]. By contrast, microencapsulated genistein, but not non-encapsulated genistein, was found to significantly reduce oxidative stress in murine colonic tissue, supporting that differences in the delivery system could explain earlier discrepancies in genistein antioxidant activity [182].

Oral administration of soyasaponin I in TNBS-induced mice was shown to significantly reduce both inflammatory markers and lipid peroxide (malondialdehyde and 4-hydroxy-2-nonenal) levels, while increasing glutathione content, SOD and catalase activity [44].

### 3.3. Myeloperoxidase (MPO) Activity

Myeloperoxidase is a peroxidase enzyme that is mostly expressed in neutrophil granulocytes and produces hypohalous acids to conduct antimicrobial activity. Elevated levels of MPO can cause oxidative damage in host tissue [222]. Reductions in MPO have been reported in various animal studies following supplementation with soy-derived bioactives. For example, oral supplementation of fermented soy germ (55% daidzein, 30% glycitein, and 15% genistein in aglycone forms for 15 days) to TNBS-induced colitis Wistar rats significantly suppressed colonic MPO compared to untreated animals [120]. In a comparable study, TNBS-induced MPO activity was suppressed in Wistar rats treated orally with genistein (100 mg/kg for 14 days) compared to untreated colitic rats [20]. Studies on TNBS-treated ICR mice fed soyasaponin Ab and I (10 mg/kg) for 5 days revealed similar results regarding MPO activity inhibition [42,44]. Reductions in MPO activity have also been reported in DSS-colitis BALB/c mice administered soybean β-conglycinin (50 or 500 mg/kg for 28 days) [71] and transported tripeptide VPY (Val-Pro-Tyr, 2-week pretreatment of drinking water with 0.1 and 1 mg/mL) [76]. Taken together, it appears that the reduction in MPO, oxidative and intestinal inflammation is attributed to various bioactive components of soy, although the exact mechanisms remain unclear.

### 3.4. Pathway Regulation

#### 3.4.1. Cytokines

Studies have shown various effects of soybean and soy derivatives on cytokine pathways, which cannot be easily integrated into a single narrative. However, decreased expression of pro-inflammatory cytokines TNF-α, IL-1β, IL-6, and IFN-y (stimulates macrophages to induce innate/adaptive immune responses) among others, have been frequently reported following administration of soy in models of inflammation with and without chemically induced colitis [42,44,77,120,168,201,213,224,225,226]. The effect of soy on anti-inflammatory cytokine IL-10 has, however, yielded variable results [131], with some studies showing increased production following administration of soy isoflavones [216], whereas others showed no effect [120,131,149]. It is possible that variability of effect reflects differences in processing, dosage, and animal model.

#### 3.4.2. Cyclooxygenase 2 (COX-2)

The pro-inflammatory enzyme COX-2 or Prostaglandin-endoperoxide synthase 2 (PTGS2) is a key inducible enzyme encoded by the PSG2 gene that is rapidly upregulated by cytokines and growth factors and is thus an important mediator of the inflammatory response. Increased COX-2 expression in colonic epithelium has been associated with IBD and DSS-colitis rodent models [102,106], with affected areas of the gut known to exhibit increased prostaglandin production [124,125].

Across the various chemically-induced colitis models reviewed, colitis induction was uniformly found to increase the mRNA expression of COX-2 in the colonic mucosa. While genistein [20], soyasaponin [42,44], and lunasin [104] have been shown to markedly attenuate colitis-induced increases in colonic COX-2 mRNA, additional studies are needed to determine potential differences in effect in terms of timing of treatment administration. For instance, the effect of genistein and soyasaponin were evaluated when administered to rodents prior to colitis induction [20,42,44], whereas lunasin was evaluated for its effect after the induction of colitis [104], for which effects were seen both at doses of 20 mg/kg and 40 mg/kg [104]. Fermented soy sauce (prepared from defatted soybeans or other protein-rich materials) was also shown to inhibit colonic COX-2 in DSS-induced C57BL/6J mice, albeit low-dose treatment (4 mL/kg) resulted in greater anti-colitic effects than that of high-dose (8 mL/kg) [225]. Of interest, COX-2 was not inhibited in DSS-treated C57BL/6 mice fed pure soy BBI (despite the anti-inflammatory effect of pure soy BBI in the DSS model), whereas in the same model, the attenuation of DSS colitis in mice fed pea seed extract containing BBIs was accompanied by significant reductions in COX-2 mRNA expression [212]. Of interest, the same study also found that pure soy BBI upregulated expression of matrix-degrading proteases, specifically metalloproteinase-14 (MMP)-14, whereas pea seed extract significantly reduced mRNA expression of MMP-2, MMP-9 and MMP-14 [212], suggesting that the actions on altered colonic immune response vary based on the dietary source of BBI.

#### 3.4.3. Toll-Like Receptors (TLRs)

Toll-like receptors are a class of proteins expressed in innate immune cells including, macrophages and dendritic cells, that recognize microbial structural components by binding to pathogen-associated molecule patterns (PAMPS). TLRs, in turn, trigger the production of pro-inflammatory mediators, including cytokines such as TNF-α and IFN-y through the NF-κB and the interferon regulatory factor 3 (IRF3) signaling pathways [227]. In this regard, TLRs play an important role in innate immune system pathways linked to inflammation, and in turn, the pathogenesis of multiple diseases involving the innate and adaptive immune systems. Indeed, mutations of the TLR are linked with chronic inflammatory conditions, including IBD [228].

Numerous studies have investigated how soy or soy bioactives mediate TLR expression and inflammation in rodent IBD models. However, these studies have varied considerably based on the type of soy-derived bioactive administered, rodent genetics, and macronutrient composition (i.e., high-fat vs. low-fat) of the underlying diet. For instance, both the soluble and insoluble fiber component isolated from soy hulls were found to block the TLR4/NF-κB inflammatory signaling pathway in DSS-treated BALB/c mice compared to control mice [229]. In another study, soy-derived BBI (50 mg/kg/day for 23 days) was shown to significantly reduce the expression of TLRs, including TLR2, TLR4, TLR6, and TLR9 in C57BL/6 mice following DSS treatment [212]. In another study, replacement of 6 or 12% of the dietary protein with isoflavone-free soy protein concentrate for 10 days was shown to blunt the DSS-induced increases in colonic IL-1β and TLR4 expression in CF-1 mice [11]. By comparison, in DSS-colitis ICR (institute of cancer research) mice, dietary supplementation with soy isoflavones (0.5% for 7 days) alleviated colitis severity and inactivated Myd88 but did not significantly alter TLR4 expression following colitis induction [149]. In a TNBS-colitis ICR mouse model, Soyasaponin Ab treatment was shown to attenuate colitis severity and inhibit NF-κB activation and expression of TLR4 [42]. In vitro, soyasaponin Ab administration to TLR4 siRNA-treated peritoneal macrophages did not affect TLR4 expression or LPS-induced NF-κB activation, suggesting that soyasaponin may ameliorate colitis through the inhibition of LPS binding to TLR4 on macrophages [42].

There is also evidence that the inflammatory potential of HFDs can be enhanced or suppressed by soy bioactives. For example, in an HFD-induced obese Sprague Dawley rat model, soy isoflavones blocked TLR4 and NF-κB expression in colonic tissue [216], whereas the long-term addition of genistein to an HFD fed to C57BL/6J mice resulted in a significant increase in TLR4 expression, as well as expression of TNF-α and IL-6 compared to controls [224].

#### 3.4.4. Peroxisome Proliferator-Activated Receptors (PPARs)

Peroxisome proliferator-activated receptors are ligand-activated nuclear hormone receptors for lipid-derived substrates. The family of PPARs plays an essential role in energy metabolism and consists of three unique members: PPAR-α, PPAR-δ, and PPAR-γ, with the latter recently elucidated for its role in bacterial-induced inflammation and the IBDs [230]. Studies investigating the effects of soy-derived isoflavones compared to that of soy protein isolate on PPAR activity have, however, yielded inconsistent findings. While some in vivo studies show PPAR-α activation due to the isoflavone content of soy [231,232], others demonstrate PPAR-α activation as a result of the non-isoflavone phytochemicals or their metabolites derived from soy [233]. With regards to PPAR-y, activation of PPAR-γ by isoflavones has been shown in vitro [231,232], whereas the in vivo effects of soy on PPAR-γ appear to be tissue-specific [234,235].

### 3.5. Microbiome

The gut microbiota is shaped by diet and plays a critical role in both the etiology and progression of IBD. Decreases in microbial diversity, as well as alterations to the *Firmicutes*: *Bacteroidetes* ratio, with reductions in beneficial taxa *Bacteroidetes*, *Lactobacillus* and *Fecalibacterium prausnitzii* have been reported in chronic inflammatory conditions including obesity and IBD [216,236,237,238]. Comparable alterations in taxa, particularly increased abundance of *Firmicutes* and decreased *Bacteroidetes*, are reported in animal models of chemically-induced colitis [212], and thus such models have been frequently used to test the in vivo effects of soy on microbiota composition.

Several studies have shown the consumption of soy protein exerts a beneficial effect on gut microbiota, leading to greater microbial diversity, decreased *Firmicutes* and increased *Lactobacillus* abundance, and enhanced bacterial production of SCFA, especially lactic and butyric acids [132,239,240]. Further, the anti-inflammatory effects of soy protein have been linked to changes in bile acid pool composition and metabolism [241,242], which itself is subject to gut microbiota modulation [243,244]. Although the interplay between the latter two factors remains unclear, bile acids act as hormone-like regulators of inflammation, and evidence suggests that dysbiosis of the gut microbiota and its reciprocal interaction with the composition and pool size of bile acids is associated with the pathophysiology of metabolic disease [245,246].

HFDs are known to result in alterations to the gut microbiota and intestinal pool of bile acids and promote inflammation [242]. In the context of an HFD, soy protein intake has been shown to promote microbiota-driven increases in bile acid transformation of primary bile acids towards their secondary forms, as well as favor reabsorption of bile acids in the colon [242]. For instance, the impairments in intestinal permeability observed in C57/BL6 mice fed an HFD composed primarily of soybean oil (40% fat, absence of isoflavones, peptides) were found to significantly correlate with increases in cecal concentrations of primary bile acids, colic, chenodeoxycholic, and alpha-muricholic acid, as well as secondary bile acids lithocholic, hyodeoxycholic, and ursodeoxycholic acid, compared to control-fed mice [242]. While these findings are consistent with previous in vitro studies demonstrating that certain bile acids, including colic acid, chenodeoxycholic, and ursodeoxycholic acid, increase tight junction permeability [247], other studies, however, have reported a protective effect both in vivo and in vitro from ursodeoxycholic and lithocholic bile acid on intestinal inflammation [248,249]. In one study, the protective effect of fermented soy on permeability and concomitant reductions in lithocholic acid occurred in supplemented rats fed either a high- or low-fat diet composed primarily of beef tallow as the fat source [217], suggesting the soy-derived components such as peptides and isoflavones can mediate the pro-inflammatory HFD-induced shift in bile acids, although this requires further study.

Some of the beneficial health effects of soybean and soy isoflavones may be attributed to their ability to stimulate or inhibit the growth of the gut microbiota population [250]. In an HFD study, the addition of 0.2% genistein significantly increased the relative abundance of *Firmicutes* (from 21.3 5 to 23.8%) and decreased Bacteroidetes (from 67.1% to 56.8%), with a concomitant increase in both *Verrucomicrobia* and *Prevotellaceae*, compared to C75BL/6 mice fed a non-supplemented HFD [224]. At the species level, these changes could be explained by shifts in *Bacteroides acidifaciens* and *Bacteroides uniformis*, whereas the genus-level changes in *Prevotella* and *Akkermansia* were mainly attributed to *Prevotella copri* and *Akkermansia muciniphila*, respectively [224]. By comparison, rats fed an HFD supplemented with soy isoflavone (150 mg/kg vs. 450 mg/kg) exhibited a significantly higher relative abundance of *Bacteroidetes* and *Proteobacteria*, and reduced the proportion of *Firmicutes* and *Firmicutes*: *Bacteroidetes* ratio [216]. In line with previous studies [251,252], the increased proportion of *Phascolarctobacterium* and concomitant decrease in *Oscillibacter*, *Morganella*, and *Pasteurella* were believed to contribute to the improvements in gut barrier integrity and reduced inflammation observed in soy isoflavone supplemented mice [216,253,254]. In another study, the addition of fermented soy to a HFD resulted in a lower proportion of Bacteroides and higher *Clostridium cluster XIVa*, as well as higher cecal acetate, butyrate, propionate, and succinate levels, compared to a HFD alone [217].

Hydrolysate-soybean media has been shown in vitro to promote a higher growth rate of both *Bifidobacteria* and *Lactobacillus* [255], whereas the addition of soy protein did not increase probiotic microorganism growth [256]. Various animal models, however, with or without colitis induction, have reported increases in abundance of anti-inflammatory taxa *Bifidiobacterium* and *Lactobacillus* [12,257,258], with improvements in the *Firmicutes* to Bacteroidetes ratio, following the feeding of soy oligosaccharides, soluble and insoluble soy-derived fibers, or soy-based products [201,229,259]. Lactic acid bacteria, such as *Lactobacillus plantarum* strains [257,258], which are known for their anti-inflammatory and antioxidant effects on IBD [257,260,261], are promoted by consuming a soy-based diet [12].

Several studies have demonstrated a protective effect from soy-based products fermented with probiotic bacteria strains on chemically-induced colitis and cancer severity. For example, fermentation of a soy-based product by *Enterococcus faecium* CRL 183 and *Lactobacillus helveticus* 416 with *Bifidobacterium longum* ATCC 15,707 significantly reduced DSS-colitis symptom severity in male Wistar rats, as well as increased abundance of *Lactobacillus* spp. and *Bifidobacterium* spp., the latter accompanied by increases in SCFA levels (propionate, acetate) compared to controls [131]. Similarly, fermentation of soy milk by *Lactococcus lactis* subsp. *lactis* S-SU2 [257], or the riboflavin-producing strain *Lactobacillus plantarum* CRL 2130 [262], has been shown to significantly reduce chemically induced colitis severity compared to those fed unfermented soy milk. Findings from the latter study suggest that riboflavin-producing lactic acid bacteria may serve as an effective anti-inflammatory therapy to promote mucosal integrity [263]. Fermented soybean pastes synthesized with probiotic species *Aspegillus oryzae*, *Bacillus subtilis-SKm*, and *Lactococcus lactis-GAm* has also been shown to inhibit both DSS-colitis and azoxymethane (AOM)-induced colon carcinogenesis [264,265].

## 4. Discussion

During the last decade, preclinical and human studies have shown a beneficial and therapeutic anti-inflammatory activity from soybean and soybean-derived compounds in chronic inflammatory disorders, including IBD. This paper reviews the preclinical evidence provided by animal modes of IBD (with or without chemically-induced colitis) that tested the role of soybean and bioactive components of soybeans on the severity of intestinal inflammation to closely examine the mechanistic effects soybean has on inflammation, oxidative stress, intestinal permeability, gut microbiota profiles, and immune systems under controlled conditions.

Our review highlights the overlapping anti-inflammatory potential of soybean and soybean bioactive compounds in experimental IBD, as well as the differences in the mechanism of action that exist due to different components within soybean components. However, our review also highlights the variability in findings between studies, which appear to depend on various factors, including rodent genetics, the method of colitis induction, as well as the duration of the feeding trial, and dosage/source/fermentation and structural composition of the soybean/soy compound/bioactive used. Other factors, including accompanying diet compounds/diet profile (e.g., HFD), quality and processing methods of soybean, and gut microbiota, could also play a role in the mechanism and effectiveness of soy to modulate intestinal inflammation.

Despite the great advancement and generation of relevant data, a limitation to note is that many studies do not report in detail the nutritional composition of the soybean compound or the diet, making it difficult to elucidate the exact role (or interaction) of soy from that of other dietary factors, and creating less reproducible experiment conditions, as previously described by our group [266]. In the future, it is important to improve reporting and that experiments are conducted in a fashion that correlates the described mechanisms, host genetics, and host microbiome.

## 5. Conclusions

Soybeans have been a major component in Asian cuisine for centuries, and soy products have increasingly become a popular and preferred choice in Western countries because of their diverse nutritional content, particularly as a high protein legume. Over the last decade, a growing body of evidence has revealed a wealth of potential health benefits from consuming soybean products, attributed primarily to the rich source of antioxidants and immunomodulatory molecules present in soy. In particular, soybean bioactives have attracted attention from a therapeutic perspective in IBD because of their anti-inflammatory, anti-oxidative, and protective effects against intestinal permeability demonstrated in rodent models of IBD. While the preclinical findings to date are promising, more studies are needed to understand and characterize the complex biochemical mechanisms through which soy bioactives interact and exert their effect, especially in the context of the genetics of the host and the microbiome of the host.

## Figures and Tables

**Table 1:** Summary of bioactives and their effect on gut inflammation.

Bioactive	Effects	Mechanisms
Phosphatidylcholine	Blocks hydrophobic bacteria and hydrophilic antigens from entering the intestine; improves mucus layer integrity and mucus secretion; ↓ oxidative stress	Mechanisms not described in detail
Soyasaponins	Antioxidant, anti-inflammatory, and immunomodulatory activity	Inhibit LPS binding to TLR4, NF-κB, and iNOS inhibition
Phytosterols	Anti-inflammatory and anti-oxidative effects; FXR antagonist (stigmasterol)	NF-κB inhibition and COX-2 downregulation
β-conglycinin and Glycin	Maintain intestinal mucosa integrity; improve epithelial cell growth; inhibit enteropathogen adhesion (*E. coli, S. typhimurium and S. enteritidis*); ↓ MPO	NF-kB/p65 inhibition
Lectin	Antibacterial, antifungal, and antiviral activities; disrupt gut barrier function; induce local inflammatory responses; ↓ immunological response; interfere with the balance of the intestinal microbiota	By binding to small bowel epithelial cells; serving as a nutrient source of bacteria; altering the gut mucosal system
Lunasin	Suppresses LPS-induced inflammatory reactions in macrophage, decrease pro-inflammatory cytokine production	Suppress PGE2 via COX-2, and NF-κB inhibition
Equol	↓ NO production; antioxidant and estrogenic activity	Inhibition of iNOS mRNA expression, ↓ NF-kB activation
Bowman-Birk Inhibitor (BBI)	Anti-inflammatory activity in the gut; suppress oxidative stress; decrease pro- IL-1β, TNF-α, IL-6, and increase IL-10 in macrophages	Inhibition of serine proteases released from inflammation-mediating cells
Soy Oligosaccharides	Benefit immune function by promoting the metabolism of beneficial commensal gut bacteria; increase levels of SOD and IgG; promote splenocyte proliferation; increase abundance in SCFA-producing bacterial taxa	Enhanced T-lymphocyte and lymphocyte proliferation
Genistein	Inhibit TNF-ɑ-induced endothelial and vascular inflammation; improve cell viability and cellular permeability; convert M1 macrophages toward the M2 phenotype	Mediation of protein kinase pathway; NF-κB inhibition; activation of the JAK signal transduction and transcription (STAT) pathway

COX-2; cyclooxygenase, JAK; Janus kinase, LPS; lipopolysaccharide, MPO; myeloperoxidase, NO; nitric oxide, iNOS; nitric oxide synthase, NF-κB; nuclear factor kappa B, PGE2; prostaglandin E2, SCFA; short-chain fatty acid, SOD; supeoxide dismutase, STAT; signal transduction and activator of transcription, TLR; toll-like receptor-4; TNFα; tumor necrosis factor-alpha.
